# DNA Repair: Dynamic Defenders against Cancer and Aging

**DOI:** 10.1371/journal.pbio.0040203

**Published:** 2006-06-13

**Authors:** Jill O Fuss, Priscilla K Cooper

## Abstract

This primer provides an introduction to mechanisms involved in DNA repair, specifically nucleotide excision repair, and their relevance to disease.

You probably weren't thinking about your body's DNA repair systems the last time you sat on the beach in the bright sunshine. Fortunately, however, while you were subjecting your DNA to the harmful effects of ultraviolet (UV) light, your cells were busy repairing the damage. The idea that our genetic material could be damaged by the sun was not appreciated in the early days of molecular biology. When Watson and Crick discovered the structure of DNA in 1953 [ 1], it was assumed that DNA is fundamentally stable since it carries the blueprint of life. However, more than 50 years of research have revealed that our DNA is under constant assault by sunlight, oxygen, radiation, various chemicals, and even our own cellular processes. Cleverly, evolution has provided our cells with a diverse set of tools to repair the damage that Mother Nature causes.

DNA repair processes restore the normal nucleotide sequence and DNA structure after damage [ 2]. These responses are highly varied and exquisitely regulated. DNA repair mechanisms are traditionally characterized by the type of damage repaired. A large variety of chemical modifications can alter normal DNA bases and either lead to mutations or block transcription if not repaired, and three distinct pathways exist to remove base damage. Base excision repair (BER) corrects DNA base alterations that do not distort the overall structure of the DNA helix, such as bases damaged by oxidation resulting from normal cellular metabolism. While BER removes single damaged bases, nucleotide excision repair (NER) removes short segments of nucleotides (called oligonucleotides) containing damaged bases. NER responds to any alteration that distorts the DNA helix and is the mechanism responsible for repairing bulky base damage caused by carcinogenic chemicals such as benzo [a]pyrene (found in cigarette smoke and automobile exhaust) as well as covalent linkages between adjacent pyrimidine bases resulting from the UV component of sunlight. NER can be divided into two classes based on where the repair occurs. NER occurring in DNA that is not undergoing transcription (i.e., most of the genome) is called global genome repair (GG-NER or GGR), while NER taking place in the transcribed strand of active genes is called transcription-coupled repair (TCR or TC-NER). We will explore NER in more detail below. Mismatch repair (MMR) is another type of excision repair that specifically removes mispaired bases resulting from replication errors.

DNA damage can also result in breaks in the DNA backbone, in one or both strands. Single-strand breaks are efficiently repaired by a mechanism that shares common features with the later steps in BER. Double-strand breaks (DSBs) are especially devastating, since by definition there is no intact complementary strand to serve as a template for repair, and even one unrepaired DSB can be lethal [ 3]. In cells that have replicated their DNA prior to cell division, the missing information can be supplied by the duplicate copy, or sister chromatid, and DSBs in these cells are faithfully repaired by homologous recombination involving the exchange of strands of DNA between the two copies. However, most cells in the body are non-dividing, and in these cells the major mechanism for repairing DSBs is by non-homologous end joining (NHEJ), which, as the name implies, involves joining two broken DNA ends without a requirement for homologous sequence and which, therefore, has a high potential for loss of genetic information.

## Hereditary defects in DNA repair.

The biological consequences of defects or deficiencies in DNA repair are varied and often severe. Mutations in genes that encode DNA repair proteins cause a wide variety of rare inherited human syndromes that exhibit diverse clinical phenotypes. Most include a premature aging phenotype, either photosensitivity or increased sensitivity to ionizing radiation exposure, greatly increased cancer risk, or all three. Nearly all hereditary DNA repair diseases are recessive, meaning that both copies of a gene must be mutated in order for the disease to develop. As a result, these diseases are extremely rare, collectively accounting for less than 5% of all human cancers [ 2]. The vast majority of human cancers are spontaneous (not inherited) and result from a combination of genetic and environmental contributions. Identifying genetic variations in the normal population that increase risk of cancer is of considerable public health interest, and DNA repair genes are likely candidates. Elucidating the molecular mechanisms that underlie inherited defects in DNA repair will provide a framework for understanding the complex patterns of predisposing genetic variations that will surely emerge from large-scale studies of spontaneous human cancers.

Diseases have been linked with defects in all types of DNA repair pathways. For example, hereditary nonpolyposis colon cancer results from defects in mismatch repair genes, and hereditary breast cancer is caused by mutations affecting the breast cancer-associated proteins BRCA1 or BRCA2 that play a role in DSB repair by homologous recombination. Here we describe in more detail the devastating human disorders known to be caused by defects in NER ( Table 1).

**Table 1:**
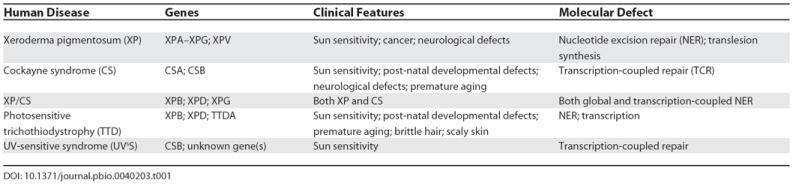
Human Diseases Caused by Defects in Nucleotide Excision Repair

## Xeroderma pigmentosum (XP).

Although rare, XP is the most common of the DNA repair defective diseases and the most well known. In the United States the frequency of XP is approximately 1 case per 250,000, and in Japan the frequency is much higher, at 1:40,000 [ 4]. The discovery of 26 XP children in one small mountain village in Guatemala [ 5] focused recent international attention on XP, but it was first described in 1874 [ 6] and was first shown to be caused by defects in NER in the late 1960s [ 7]. The most evident clinical features of XP are extreme sun sensitivity with marked thickening of the skin (“xeroderma”), together with changes in pigmentation (“pigmentosum”) and a very high incidence of skin cancers on sun-exposed regions of the body. Eye and neurological abnormalities are also common. XP patients can be subdivided into eight complementation groups, XP-A through -G plus XP-V, based on which gene is affected. Seven of the eight genes *(XPA* through *XPG)* are directly involved in NER, while the *XPV* (variant) gene product is a DNA polymerase that is involved in DNA replication past UV lesions (“translesion synthesis”).

## Cockayne syndrome (CS).

First described by Edward Cockayne in the 1930s [ 8], CS is a developmental disorder involving profound mental retardation, premature aging, and severe wasting that become evident within the first few years of life and lead to death in childhood. CS patients are also sun-sensitive, but unlike XP patients, do not develop skin cancers. CS most frequently arises from mutations in genes encoding the CSA and CSB proteins. Both proteins are required for TCR, suggesting that defective repair of lesions in active DNA may be causative for the disease. However, the exact cellular function of the CSB protein has not yet been determined, and the nature of its causative role in CS is further complicated by the surprising finding that a patient diagnosed with UV-sensitive syndrome, an extremely mild sun-sensitive disease without any of the severe clinical features of CS, completely lacks any CSB protein [ 9]. In rare cases, certain mutations in the *XPB*, *XPD*, and *XPG* genes lead to XP combined with CS. These XP/CS patients are profoundly affected, having clinical features of both disorders.

## Trichothiodystrophy (TTD).

This syndrome is characterized by sulfur (“thio”)-deficient brittle hair (“tricho”) with malformed (“dystrophy”) hair shafts, fish-like scales on the skin, mental and physical retardation, and sun sensitivity in some complementation groups [ 10]. Mutations in the *XPD* and *XPB* genes have been found to cause the sun-sensitive form of TTD [ 11, 12], but, until recently, the molecular basis for a third sun-sensitive TTD complementation group, TTD-A, was unknown. In 2004 the gene mutated in TTD-A was identified and shown to encode a tiny protein of only 71 amino acids (8 kDa) [ 13]. Remarkably, the small TTDA protein is the tenth subunit of a key multi-protein complex called TFIIH that was first described as a transcription factor essential for transcription initiation by RNA polymerase II (RNAPII) [ 14] and was later found to be required for NER as well [ 15]. The XPB and XPD proteins are also components of TFIIH, making it clear that compromised TFIIH defines the molecular basis for the sun-sensitive form of TTD.

## Nucleotide excision repair.

**Figure 1:**
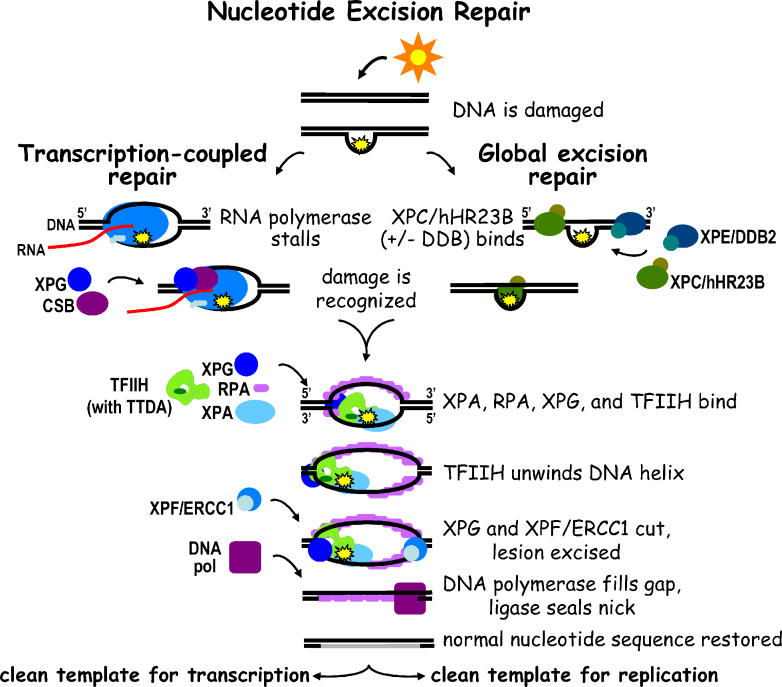
Nucleotide Excision Repair Schematic When DNA is damaged by sunlight, the damage is recognized differently depending on whether the DNA is transcriptionally active (transcription-coupled repair) or not (global excision repair). After the initial recognition step, the damage is repaired in a similar manner with the final outcome being the restoration of the normal nucleotide sequence. A more detailed description is provided in the text.

Much of our understanding of NER in humans has come from studies of cells from XP and CS patients. NER (shown schematically in Figure 1) can be generally broken down into five steps: damage recognition to initiate repair, helix opening and unwinding, incision on either side of the lesion, excision (release) of the damage-containing oligonucleotide, and DNA synthesis and ligation. GG-NER and TCR differ in how damage is recognized and also in biological outcome. GG-NER protects against mutations in the genome from replication of unrepaired lesions that could ultimately lead to cancer, while TCR ensures that genes are transcribed correctly and efficiently, a function that is now appreciated to be important in protecting against aging [ 16].

The NER machinery responds when DNA suffers damage that distorts its helical structure. DNA lesions caused by UV are the best example of this type of damage and have been extensively studied, since they are easy to generate in the lab, are stable in DNA, and are biologically important for any organism that is exposed to sunlight. Repair is initiated when the helix distortion is recognized by the XPC protein together with its partner hHR23B. Some less distorting lesions first require initial recognition by the DDB complex, which is mutated in XP-E cells. The TFIIH complex is then recruited by XPC and is immediately joined by the XPA protein, the single-stranded DNA binding protein RPA, and the XPG protein. The XPB and XPD components of TFIIH are DNA helicases (named for ability to disrupt the double helix), and through their action TFIIH unwinds the DNA surrounding the lesion until a 30-nucleotide “bubble” is formed. RPA and XPA stabilize the DNA bubble and also help to position two endonucleases at the bubble junctions, where they serve as “scissors” to cut out the DNA damage. The first incision, on the 3′ side of the bubble relative to the lesion, is made by XPG, which also coordinates the second incision on the 5′ side of the bubble by the XPF protein and its partner ERCC1 [ 17, 18]. A lesion-containing DNA fragment of 25–32 nucleotides is released, the gap is filled in by a DNA polymerase using the information from the intact complementary strand, and the remaining nick is sealed by a DNA ligase that restores the intact strand.

A major decision point in NER concerns how the enzymatic machinery “knows” which of the two DNA strands should be cleaved, or indeed whether cleavage should occur at all (which is appropriate only if a lesion is actually present). Presumably some mechanism for lesion verification must be involved. The damage-binding protein XPA is likely to play a role, since it is essential for NER incisions. However, as described below, new evidence suggests the possibility that the multi-functional TFIIH complex is also involved.

## Transcription-coupled repair.

DNA that is undergoing transcription initiates NER in a different manner ( Figure 1), since UV lesions directly block elongating RNAPII [ 19, 20]. The original and still current model for TCR [ 21] postulates that RNAP blocked by a lesion in the DNA template is more efficiently recognized than the lesion itself, providing a potent signal for recruiting repair enzymes. This idea nicely explains how TCR occurs much more rapidly than GG-NER [ 21]. Consistent with different recognition signals, the XPC/hHR23B protein complex is not required for TCR [ 22, 23], and instead the CSB protein has been implicated in initiation of TCR through recognition of stalled RNAP [ 24], probably in concert with XPG [ 25]. After the recognition step, however, it is not clear how the recruited DNA repair proteins gain access to the DNA lesion, since it is occluded by the stalled polymerase [ 26]. Backup of RNAP facilitated by the transcription elongation factor TFIIS is one proposed mechanism for removing the polymerase from the lesion [ 27]; however, CSB actually appears to prevent TFIIS action [ 28, 29], and hence backup may be an alternative to TCR. Degradation of the arrested RNAP has also been proposed, but this is likely to be a last resort when TCR fails, rather than part of the mechanism [ 30]. Recent evidence suggests that TFIIH in the presence of XPG may function to remodel RNAPII in an ATP-dependent manner during TCR through large-scale conformational changes that allow access to the lesion without removal of the polymerase [ 25]. In any case, the subsequent steps of TCR evidently proceed in a manner similar to GG-NER, with the final outcome being the efficient generation of a lesion-free template for transcription.

## TFIIH in transcription.

In addition to TCR, transcription and NER are linked by the separate involvement of TFIIH in both processes. In transcription initiation, TFIIH is required for unwinding the DNA helix at the transcription start site, and XPB is essential for this process. TFIIH also has kinase activity provided by its CAK kinase three-protein subunit, which phosphorylates the carboxyl-terminal domain (CTD) of RNAPII. CTD phosphorylation is required for the polymerase to escape the promoter and begin elongating the transcript. How TFIIH is shared between NER and transcription is an open question. Innovative imaging techniques have begun to answer this question by probing the dynamics of TFIIH in living cells.

## Cellular dynamics of DNA repair.

Live-cell imaging is a powerful technique to probe the cellular responses of the NER machinery to DNA damage, and it has been used to demonstrate that the NER proteins do not appear to exist in a pre-assembled complex but rather assemble in an ordered fashion at sites of DNA damage [ 31]. In particular, the dynamic movements of the multi-functional TFIIH have been monitored in living cells by linking a TFIIH subunit to the green fluorescent protein (GFP) from jellyfish. After photo-bleaching a portion of the cell's nucleus with a laser, a population of fluorescently labeled proteins can be seen moving back into the bleached area over time, and the speed at which this repopulation takes place can be measured. Hoogstraten et al (2002) used live-cell imaging of GFP-labeled XPB to examine the crosstalk of TFIIH between transcription and repair and showed that TFIIH migrates freely in the nucleus, functioning in repair or transcription with different kinetics [ 32]. TFIIH molecules interact longer with NER sites than with transcription sites (5 minutes versus 10 seconds). This simple observation provided significant insight into the origin of TTD phenotypes. TTD-A cells were known to have low levels of TFIIH, but TFIIH isolated from these cells had normal in vitro enzymatic activities, suggesting that TFIIH stability rather than function might be compromised, and indeed the TTDA protein increases TFIIH stability [ 13]. The live-cell imaging result provided a framework to interpret this observation. Since TFIIH molecules are recycled much more rapidly during transcription than during NER, higher concentrations of TFIIH are required to efficiently carry out NER. Thus low levels of TFIIH have a more dramatic effect on NER than on transcription and would cause an apparent defect in transcription only in terminally differentiated tissues with high transcriptional loads such as hair and skin, which is what is observed in TTD patients.

In this issue of *PLoS Biology*, Giglia-Mari et al. used live-cell imaging to explore the dynamic movements of TTDA and XPD [ 33]. TTDA (and XPD to a lesser extent) was shown to be present in two populations that move with different dynamics: one population was stably associated with TFIIH (slow moving), while the other population moved freely around the cell (fast moving), even shuttling between the nucleus and the cytoplasm. After UV irradiation, more of the TTDA was found to be slow moving, suggesting that TTDA particularly remains associated with TFIIH during NER. The authors established that in fact TTDA stably associates with TFIIH only during productive NER events by using a clever trick. They treated the cells with actinomycin D (ActD), a chemical that inserts into DNA and then damages it when exposed to blue light but that is *not* repaired by NER. The initial responders in NER, XPC, and TFIIH (monitored with XPB-GFP), were recruited to the ActD lesions, but not TTDA or the later NER factors, XPA or ERCC1. TTDA was not fooled by the ActD lesions, suggesting that TFIIH—and possibly TTDA itself—plays a role in lesion verification and the recruitment of subsequent NER factors. Two other recent observations also suggest involvement of TFIIH in lesion verification, perhaps involving XPB and/or XPD. The altered TFIIH in cells from XP-D/CS patients results in aberrant production of incisions at sites of transcription rather than at DNA lesions [ 34], and a recent crystallographic structure of an XPB homolog has revealed the surprising existence of a domain that recognizes damaged DNA [ 35].

## Perspectives.

**Figure 2:**
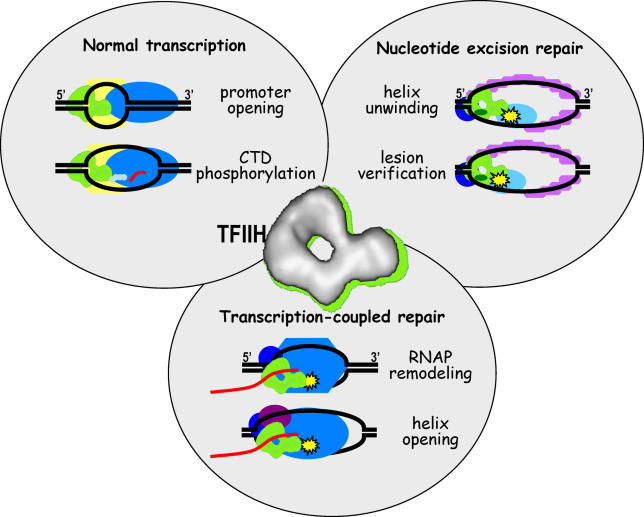
TFIIH Is a Multi-Functional Protein Complex TFIIH participates in normal transcription, NER, and TCR and has multiple functions in each of these processes. A medium-resolution electron microscopy structure of the human TFIIH complex [ 36] shows that TFIIH forms a ring-like structure with a protrusion that contains the CAK kinase complex.

The TFIIH complex sits at the crossroads of GG-NER, transcription, and TCR ( Figure 2). It has two distinct functions in initiation of transcription and a still-expanding number of different functions in NER, certainly including opening of the helix to allow incision but perhaps also including lesion verification and even remodeling of the RNAP in TCR. Different defects in its multiple functions cause three different human syndromes: XP, XP/CS, and TTD. Determining the precise mechanisms of each of these functions will require high-resolution structural information of TFIIH components and complexes, coupled with innovative biological experiments such as those that probe the dynamic movements of TFIIH within the cell. Such future investigations will provide further key insights into the molecular basis of human repair deficiency diseases that result in cancer predisposition and premature aging.

## Abbreviations

ActD: actinomycin D
BER: base excision repair
CS: Cockayne syndrome
DSB: double-strand break
GFP: green fluorescent protein
GG-NER: global genome repair
MMR: mismatch repair
NER: nucleotide excision repair
RNAPII: RNA polymerase II
TCR: transcription-coupled repair
TTD: trichothiodystrophy
UV: ultraviolet
XP: xeroderma pigmentosum
